# Cold season dose rate contributions from gamma, radon, thoron or progeny in legacy mines with high natural background radiation

**DOI:** 10.1093/rpd/ncad178

**Published:** 2023-06-19

**Authors:** Hallvard Haanes, Sven Dahlgren, Anne Liv Rudjord

**Affiliations:** Norwegian Radiation and Nuclear Safety Authority, P.O. box 55, 1332 Østerås, Norway; Vestfold and Telemark County Council, Fylkeshuset, Svend Foynsgt. 9, 3110 Tønsberg, Norway; The Njord Centre, Departments of Geosciences and Physics, University of Oslo, 0313 Oslo, Norway; Norwegian Radiation and Nuclear Safety Authority, P.O. box 55, 1332 Østerås, Norway

## Abstract

In areas with high natural background radiation, underground cavities tend to have high levels of airborne radionuclides. Within mines, occupancy may involve significant exposure to airborne radionuclides like radon (^222^Rn), thoron (^220^Rn) and progeny. The Fen carbonatite complex in Norway has legacy mines going through bedrock with significantly elevated levels of uranium (^238^U) and especially thorium (^232^Th), and significant levels of their progeny ^222^Rn and ^220^Rn. There are also significantly elevated levels of gamma radiation in these mines. These mines are naturally chimney ventilated and release large volumes of air to the outdoors giving a large local outdoor impact. We placed alpha track detectors at several localities within these mines to measure airborne radionuclides and measured gamma radiation of bedrock at each locality. The bedrock within the mines shows levels up to 1900 Bq kg^−1^ for ^238^U, 12 000 Bq kg^−1^ for ^232^Th and gamma dose rates up to 11 μSv h^−1^. Maximum levels of airborne radionuclides were 45 000 Bq m^−3^ for ^220^Rn and 6900 Bq m^−3^ for ^222^Rn. In addition, we measured levels of thoron progeny (TnP). In order to estimate radiation dose contribution, TnP should be assessed rather than ^220^Rn, but deposition-based detectors may be biased by the airflow of mine-draft. We present dose rate contributions using UNSCEAR dose conversion factors, and correcting for airflow bias, finding a combined cold season dose rate within these mines of 17–24 μSv h^−1^. Interestingly, fractional dose rate contributions vary from 0.02 to 0.6 for gamma, 0.33 to 0.95 for radon and 0.1 to 0.25 for TnP.

## Introduction

Radon (^222^Rn) and thoron (^220^Rn) originate from radium isotopes (^226^Ra and ^224^Ra), respectively, in the uranium (^238^U) and thorium (^232^Th) decay series. Mining activity in areas with elevated naturally occurring background radiation is therefore often associated with radiological issues with these radioactive gasses^(1–3)^. Dose is given by the progeny of ^222^Rn (RnP) and ^220^Rn (TnP) that deposit in the respiratory tract^(4–6)^. Within uranium mines, levels of ^222^Rn and RnP can be very high^(7, 8)^, and the health risk to miners has been well documented^(2, 9, 10)^. Because of ^220^Rn half-life (55 s), measurements and equilibrium with TnP are uncertain^(11–13)^ and dose rates should be based on TnP measurements^(14–17)^. For legacy mines within bedrock rich in ^232^Th, it has been shown that the level of TnP can be high (2.4–2.9 WL)^(3)^ and high levels of ^220^Rn (40 KBq m^−3^) naturally chimney ventilate^(1)^. Knowledge about variation of ^222^Rn, ^220^Rn and TnP within such mines and natural ventilation and exhalation to the surface is thus needed.

**Figure 1:**
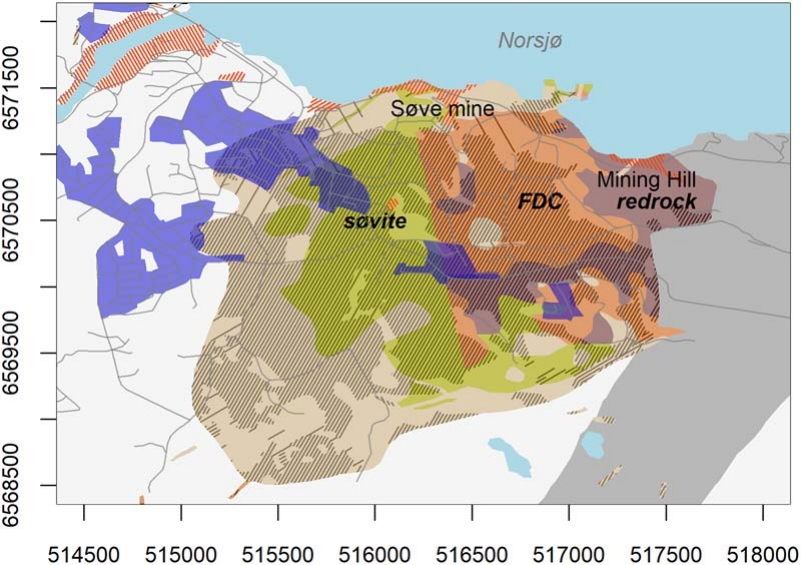
Map of Fen igneous complex with radioactive bedrock and the two legacy mining sites (Søve mine and Mining Hill) but see also Dahlgren^(19)^ for an updated and more detailed distribution of FDC. Axes give UTM coordinates (32N). Holocene deposits in gray hachure, anthropogenic deposits in orange hachure and densely populated areas in blue.

**Figure 2:**
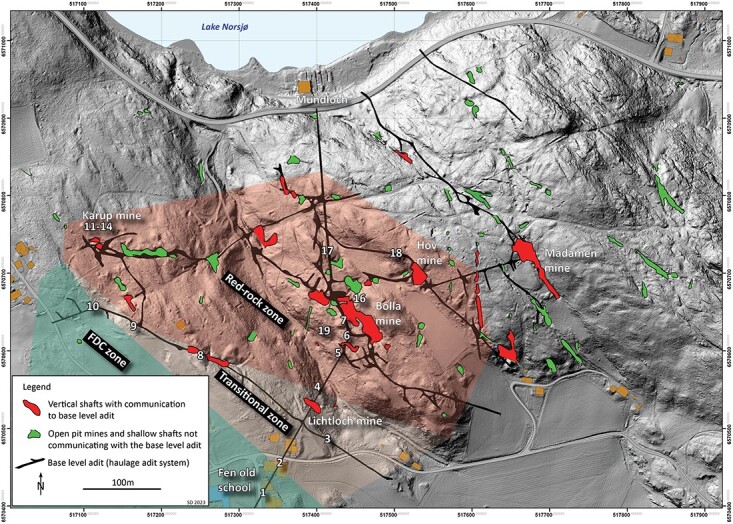
Laser imaging, detection and ranging (LIDAR) map of Fen iron legacy mines (Mining Hill) with open pits in green and mine openings in red and measurement points given by numbers. Numbers 1 and 2 are the SM, 3–6 Lichtloch, 7 and 19 Bolla mine, 8 and 9 the AC mine, 10 the western mine, 11–14 the Karup mine and 16–18 the main adit. Main adit base level runs from Fen Bay to beneath Gruvehaugen (large mine Bolla opening in center of map) and all mines below this level are flooded with water. Bedrock type is shown as red for redrock, green for FDC and the transition zone in between.

In the Fen igneous complex in Norway (Figure 1), legacy mines run through bedrock where background radiation is elevated for radionuclides in the decay series of ^238^U and especially ^232^Th^(3, 18)^. In the eastern part of the complex and running through redrock, iron legacy mines older than 100 years are found in Mining Hill (Figure 2). In the western part, the Søve legacy niobium mine, also called Tuftestollen, from the 1960s runs through mainly søvite but also Fe-Dolomite carbonatite (FDC), formerly known as rauhaugite^(19)^. Redrock activity concentrations range up to 12 000 Bq kg^−1^ of ^232^Th and søvite range up to 1400 Bq kg^−1^ of ^226^Ra^(3)^. The natural chimney ventilation of the iron legacy mines involves a seasonal direction of mine draft with sinking airflow in summer and cold air flowing out of low lying mine-openings. In winter, the mine draft is warmer than outside air and ventilates up through higher lying mine openings^(1)^. FDC has intermediate levels of radioactivity^(3)^ and is because of its contents of rare earth elements (REE) attractive for new mining activities. Regarding any occupancy in existing mines or future ones because of REE mining in FDC and søvite, dose rate pathways should be assessed for gamma radiation, radon and thoron progeny in such mines. Since mine draft has opposite directions between seasons, the air at different places within mines may differ between seasons, and exposure pathways must be assessed for each season. Here we have assessed the winter season with a mainly rising mine draft and the exposure pathways of ^222^Rn, ^220^Rn and TnP within the legacy iron mines (including one adit in FDC) and in the niobium legacy mine.

## Methods

### Study area and measurement points

The Fen complex (59.275°N, 9.304°E) contains bedrocks with different background radiation. Redrock in the eastern part (Figures 1 and 2) has especially elevated levels of ^232^Th and was mined because of associated iron ore (hematite). Activity concentrations range from 670 to 12 000 Bq kg^−1^ of ^232^Th and from 44 to 550 Bq kg^−1^ of ^238^U, involving exhalation rates of 2–25 Bq s^−1^ m^−2^ for ^220^Rn and 0.008–0.08 Bq s^−1^ m^−2^ for ^222^Rn^(3)^. By comparison, søvite in the western part (Figure 1) was mined because of niobium ore and has levels from 10 to 780 Bq kg^−1^ of ^232^Th and from 10 to 1400 Bq kg^−1^ of ^226^Ra^(3, 20)^. However, the niobium mine also run through sections of FDC and it should be noted that the søvite bedrock is very heterogenous with regard to distribution of elements and radionuclides^(21)^. FDC is otherwise found in the middle of Fen complex (Figure 1) with a transition zone toward redrock (Figure 2)^(19)^. FDC has levels from 160 to 7000 Bq kg^−1^ of ^232^Th and from 20 to 290 Bq kg^−1^ of ^226^Ra^(3, 20)^. FDC is also enriched in REE, which is attractive for new mining activities (see Reference^(19)^ for more details).

**Figure 3:**
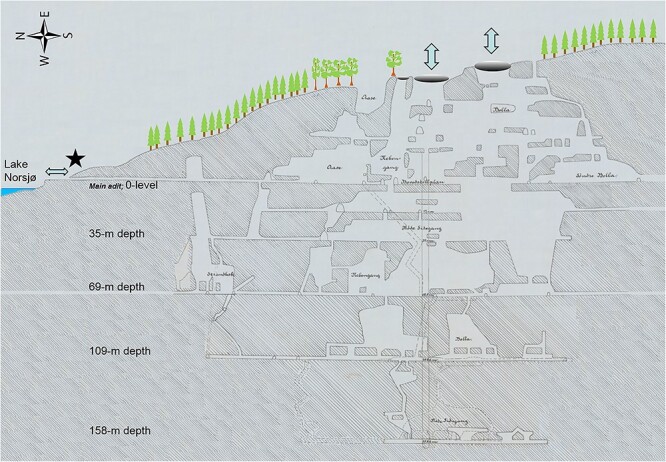
Cross section of the iron legacy mines rewritten from historical records^(43)^, illustrating the large mine openings on the top of in Mining Hill and natural chimney ventilation through these and lower openings^(1)^.

The Mining Hill iron legacy mines form a complex system with open pits and mine openings running through mainly red rock with iron ore (Figures 2 and 3). The natural chimney ventilation of the iron legacy mines involves large volumes of air ventilating with high activity concentrations. Seasonal directions of the mine draft are mainly upwards in winter and mainly downwards in summer but variable in spring and autumn because of diurnal variations of outdoors temperatures^(1)^. Approximate air temperatures within the iron mines are 4–6°C, which explains the main chimney ventilation with a rising draft in winter months with outdoor sub-zero temperatures^(1)^. The one-adit Søve niobium mine has two openings, one main and one higher lying opening, and field observations suggest natural chimney ventilation where the draft direction changes between seasons, being inwards from the entrance opening in winter.

The legacy iron mines at Mining Hill are dangerous with rock fall and are difficult to access, with climbing equipment being required at several places because of vertical shafts. This puts restrain on fieldwork within these mines, both regarding the number of possible entries and on how long one can stay. Therefore, measurements are limited and alpha track detectors, which are fast to deploy and collect, were used. The detectors were hung from the roof and the distance to the wall was recorded. The Søve niobium mine is one-adit and was used both to mine ore and for transport. Much of the mine system within the legacy iron mines was used for transport and would normally be termed haulage adits and haulage shafts, respectively, as for the main adit and the Bolla shaft. We chose localities spread out through much of this haulage system and these will be termed adits and shafts within this paper. Localities 1–19 are within Mining Hill. Of these, only the Hov mine has a true mine gallery, but ore was also taken out from Karup shaft. Only locality nr 1 is within the FDC zone, and it was by far the driest and most dusty locality, probably because of a combination of the near absence of cracks in the bedrock for ground water to enter and because this adit is far away from any shaft where surface water enters. At all other localities the air was damp, and water was dripping from the roof and walls at different magnitudes. The main transport (haulage) adit of Mining Hill runs from, and at level with, Norsjø and inwards with lower levels flooded by water. Localities were (Figure 2):

1. Fen School adit; near its dead end.

2. Fen School adit, 50 m W of the crossing with Anne Cathrine (AC) adit.

3. Lichtloch (L) shaft base at crossing with L adit.

4–6. Lichtloch cross-cut adit at 54, 106 and 129 m E of the crossing with AC adit; transport (haulage) between western and central (Bolla) parts of Mining Hill. Nr 6 is close to the Bolla shaft and rich in iron ore.

7. Bolla shaft. Iron ore was taken out in Bolla adit which crosses this shaft, which was also used for transport.

8. AC adit at shaft base.

9. AC adit at crossing with the western adit (below).

10 Western adit, which is the northwestern most part of the adit system, and which belongs to the adit system between the Karup and AC shafts.

11–14 Karup mine shaft, which is the shaft down to the northern end of the adit leading to the AC shaft.

16–17. Main adit transport (haulage) system between Bolla mine shaft and Mundloch (Nr 16, weakly inclined).

18. Hov adit; crossing near the Hov mine gallery, which is too dangerous to enter. The Hov mine gallery is connected by a branch of the adit, which joins the main adit near Mundloch (the main mine opening near Lake Norsjø.

19. Bolla-Bolladalen cross-cut; early transport (haulage) adit, ~50 meters above main adit (nr 16–17) and ca 24 m beneath surface. Short and with two entrances, and in such a way, standing out from the other localities above.

### Gamma measurements

**Figure 4:**
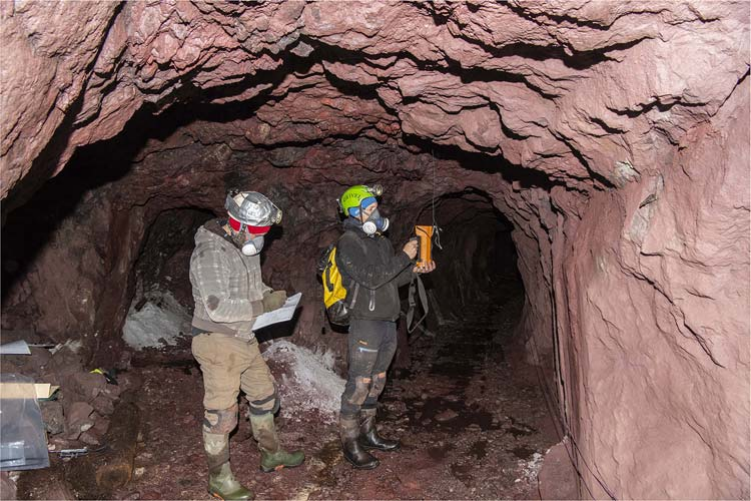
Photo of one locality, showing alpha track detectors as hung from the roof and the position of the GEORADIS gamma spectrometer.

Measurements of gamma radiation within the legacy mines were done through gamma spectrometry using a Georadis GT-30 held at around 1 meter height at the position of the alpha track detector, directed toward the roof (Figure 4). Six were at localities within a transition zone of mixed bedrock between the redrock and the FDC zone in Mining Hill (nr 2–4 and 8–10, Figure 2), one within the FDC zone (nr 1), 11 measurements at localities within the redrock zone (nr 5–7, 11–14 and 16–19) and one within the søvite zone (nr 20). The instrument measures ^214^Bi and ^228^Ac and, assuming secular equilibrium, gives the concentrations of total uranium (where the main fraction is ^238^U but 0.72% is ^235^U) and ^232^Th in bedrock, respectively, as well as an estimate of the ambient gamma dose rate (μSv h^−1^). For the typical activity concentrations of Fen bedrock, uncertainties (1 sigma) in gamma measurements are for ^238^U up to 10% at the lowest activity concentrations but below 1% at mean bedrock levels, and for ^232^Th up to 17% at the lowest measurements but below 2% at mean bedrock levels. Gamma measurements were made at all localities where alpha track detector measurements were done.

### Alpha track detector measurements

To assess variation in levels of ^222^Rn, ^220^Rn and TnP within these legacy mines, short-period time-integrated measurements were done with alpha track detectors at different mine localities at known distances from the walls. These were exposed from 27 to 28 November in the western part of Mining Hill (Figure 2: Locations 1–14) and from 5 to 6 December at in the eastern part of Mining Hill (Figure 2: Localities 16–19) and within Søve niobium mine. Exposure time varied from 18 to 30 h per alpha track detector, averaging 27 h in November and 21 h in December. Detectors hung at distances ranging from 40 to 150 cm (median: 60, mean: 69, SD: 31) from the nearest wall (source). The airflow at each locality was estimated qualitatively from observation of a ca 1 meter long, thin plastic strip hanging from the roof, fluttering more or less, indicating the direction and speed of the draught.

Levels of ^222^Rn and ^220^Rn were measured at all assessed mine localities with a pair of diffusion chamber solid-state nuclear track detectors (SSNTD) (Radtrack2 CR-39). One of these only lets in and measures ^222^Rn, whereas the second detector of the pair has holes for measurement of both ^222^Rn and ^220^Rn. The difference in track density between the two detectors is used to estimate ^220^Rn. These were provided and analysed by Radonova Laboratories (www.radonova.no). Because of the very short exposure time compared with the 3-month measurement time for which these detector measurements are calibrated, and because very high levels of ^220^Rn only affect the detector with holes but not the detector without holes during the short exposure time, detection limits (MDA) were higher than the calculated activity concentration in 15 of 20 detector pairs. For ^220^Rn, we therefore used the calculated values (based on alpha track numbers). Uncertainties (1 SD) ranged for ^222^Rn from 13 to 100% (median: 50%) and for ^220^Rn from 9 to 49% (median: 35%).

December measurements at some Mining Hill localities and in the niobium mine included TnP deposition detectors with an SSNTD covered by a Mylar film so only ^212^Po among TnP has sufficient energy to penetrate. The National Institute of Radiological Sciences (Japan) developed these detectors, laboratory calibrated total deposition rate of TnP^(22)^ and analysed the exposed detectors. Within mines there is often a draft and there may be an upward bias on deposition velocities from air movement^(23, 24)^. Even so, one study from a mine with drafts up to more than 0.4 m s^−1^ that used deposition-based detectors got similar EECRn values from these as measured with in situ instruments^(25)^. Normally, indoor calibration is done at 0.02 m s^−1^ and it has been shown that increasing ventilation rates (modest airflow) involve a bias with an approximate linear increase in deposition velocity^(26, 27)^. An outdoors study at even higher airflow velocities confirms the bias but suggest it may be too stringent to standardise by dividing the detector-estimated EEC thoron value with the ratio between airflow at laboratory calibration and measured wind velocity, since airflow velocity can be lower and must be measured along the detector surface^(28)^. This latter study used a statistical model to demonstrate the deposition detector bias from airflow but also identified the simultaneous importance of variation in ^220^Rn and ^222^Rn. The study showed that a mean airflow velocity along detectors of 0.2 m s^−1^ involved a mean bias from airflow that comprised a 0.87 fraction of the measurement (SD = 0.05).

The data used in this study can be made available at request to the corresponding author.

### Dose rate contributions from ^222^Rn, ^220^Rn and TnP

We apply the UNSCEAR dose conversion factors (DCF) for ^222^Rn and ^220^Rn progeny of 9 and 40 nSv (Bq m^−3^ h)^−1^, respectively^(12, 13)^. For ^222^Rn, we assume an equilibrium factor, F, like the average found in a manganese mine of 0.36^(29)^, and for comparison, we calculate the ^220^Rn cold season dose rate contribution using an *F*-value of 0.04 for a centered position in the wolframite mine^(25)^. ^220^Rn measurements involve large uncertainties and dose rates should rather be calculated from TnP^(14–17)^. We also calculate the dose rate contribution from TnP, but regarding mine draft, consider both dose rates potentially biased by the mine draft as well as dose rates corrected for mine draft assuming a 0.87 fraction of airflow bias from the mine draft.

### Calculations, statistics, weather and maps

Calculations and statistics were performed with R^(30)^. TnP values are expressed as equilibrium equivalent concentrations for ^220^Rn (i.e. EECTn), and the equilibrium factor, F, was calculated as the ratio between these. Bedrock was grouped according to main bedrock, but with differentiation between redrock localities with or without iron ore. Hourly weather data were downloaded (www.eklima.no) for the deployment periods from a nearby weather station (St.no = 32060, 59.380644°N, 9.201890°E). Map layers of bedrock and loose masses (Holocene and anthropogenic deposits) were downloaded from the Geological Survey of Norway and map layers of populated areas et cetera were downloaded from Kartverket (all via www.kartkatalog.geonorge.no). The map in Figure 1 was made in R^(30)^, using packages sp^(31)^ and rgdal^(32)^.

## Results

Gamma spectrometry showed gamma radiation dose rates ranging from 0.1 to 11 μSv h^−1^ (median: 4.3, mean: 5.1, SD: 3.6) and bedrock activity concentrations ranging from 34 to 1900 Bq kg^−1^ (median: 930, mean: 1000, SD: 560) of ^238^U and from 28 to 12 000 Bq kg^−1^ (median: 4900, mean: 5700, SD: 4200) of ^232^Th. Variation among localities were up to 4-fold for ^238^U, 20-fold for gamma and 40-fold for ^232^Th, with the niobium mine standing out with lowest concentrations (Table 1). We notice increasing levels of gamma radiation and ^232^Th toward redrock in the eastern part of Mining Hill (Figures 1 and 2 and Table 1). In redrock with iron ore, the levels of gamma and ^232^Th were highest with means of 8.1 (SD: 2.0) μSv h^−1^ and 9300 (SD: 2400) Bq kg^−1^, and a ^238^U mean of 1300 (SD: 300) Bq kg^−1^. In redrock without ore, these means were similar for gamma radiation and ^232^Th (8.0 (SD: 2.2) μSv h^−1^ and 9000 (SD: 2500) Bq kg^−1^), whereas ^238^U was somewhat higher (1500 (SD: 480) Bq kg^−1^). In the FDC zone even lower gamma radiation, ^232^Th and ^238^U were found (1.3 μSv h^−1^, 1300 and 360 Bq kg^−1^, respectively). In the transition zone, intermediate means were found (2.6 (SD: 0.5) μSv h^−1^, 2700 (SD: 720) and 910 (SD: 290) Bq kg^−1^, respectively). Notice the low gamma dose rate contributions in the niobium mine (Søve) because of the low ^232^Th level here (Table 1).

**Table 1:** Gamma radiation (*γ*) dose rate (DR, μSv h^−1^) and bedrock content (Bq kg^−1^) of ^238^U and ^232^Th in Fen legacy iron mine localities and Søve Niobium mine. Type of bedrock is given per measurement site (MS) but also the main one per adit or shaft is given (zone). Fe-Dolomite carbonatite is abbreviated FDC. In Karup shaft, the locality name is marked with number of meters below surface of the measurement point. Control situated in School office in area is indoors and above Holocene clay deposits (HCD). Abbreviations Karup: K, School mine: SM.

Nr	Mine	Type	Zone	MS bedrock	^238^U (Bq kg^−1^)	^232^Th (Bq kg^−1^)	*γ* DR (μSv h^−1^)
1	SM	adit	FDC	FDC	360	1300	1.3
2	SM	adit	transition	redrock	740	2700	2.5
3	Lichtloch	shaft	transition	redrock/FDC	890	2200	2.2
4	Lichtloch	adit	transition	FDC/^232^Th veins	900	2000	2.1
5	Lichtloch	adit	redrock	redrock	1100	5400	4.9
6	Lichtloch	adit	redrock	redrock iron ore	1200	11 000	9.1
7	Bolla	shaft	redrock	redrock iron ore	1500	11 000	9.9
8	AnneCat	adit^a^	transition	FDC/some redrock	1500	2200	2.6
9	AnneCat	adit	transition	redrock	680	3500	3.1
10	Western	adit	transition	redrock/FDC	780	3700	3.3
11	K-40 m	shaft	redrock	redrock iron ore	1100	9200	7.9
12	K-26 m	shaft	redrock	redrock iron ore	1600	11 000	9.4
13	K-17 m	shaft	redrock	redrock iron ore	1400	8800	7.8
14	K-6 m	shaft	redrock	redrock iron ore	800	4900	4.3
16	Main adit	adit^b^	redrock	redrock	1800	9000	8.1
17	Main adit	adit	redrock	redrock	1900	11 000	9.4
18	Hov	adit	redrock	redrock	940	8400	7.2
19	Bolla	adit	redrock	Redrock/FDC	1900	12 000	11
20	Søve	adit	søvite	fenite/some søvite	330	300	0.49
15	Control	Office		HCD	34	28	0.06

a At base of shaft.

b Inclined adit.

**Figure 5:**
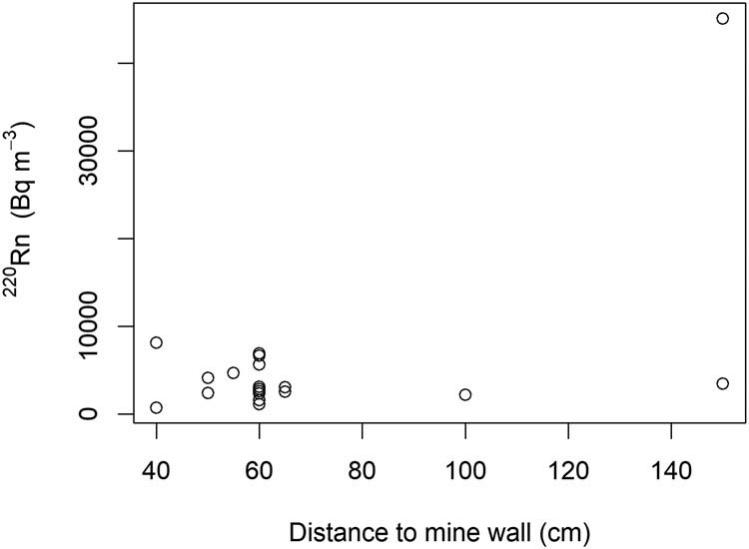
Level of ^220^Rn measured at different localities within Fen iron mines plotted against the distance to mine wall for each alpha track detector (see text for correlation).

**Figure 6:**
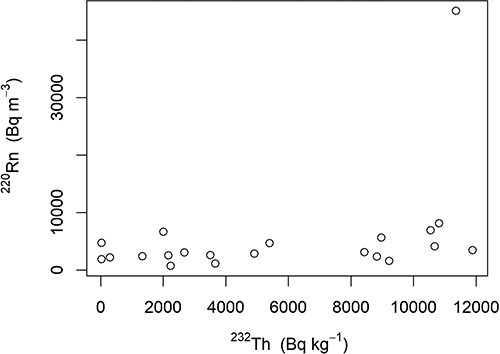
Level of ^220^Rn measured at different localities within Fen iron mines plotted against measured level of ^232^Th in the bedrock of the mine wall (see text for correlation).

Outside temperatures ranged during the November deployment from −9 to −3°C (mean: −6°C) and during the December deployment from −6 to −1°C (mean: −4°C). During both periods the observed mine draft within the mines was rising. Outdoor wind velocities ranged from 0.3 to 2.8 m s^−1^ (mean: 1.2 m s^−1^) mainly from northeast but also from northwest. The activity concentrations in mine air for ^222^Rn ranged from 500 to 6900 Bq m^−3^ (median: 1300, mean: 2000, SD: 2100) and for ^220^Rn from 700 to 45 000 Bq m^−3^ (median: 3100, mean: 5500, SD: 9300). There was some variation among localities for ^222^Rn but especially for ^220^Rn (Table 2). Especially in the niobium mine (Søve) and in the Hov and western minet in Mining Hill, ^222^Rn was high. Levels of ^220^Rn were markedly higher at localities within redrock with iron ore but with large variation (mean: 11 000, SD: 17 000 Bq m^−3^). Interestingly, levels of ^220^Rn were much lower in localities with redrock without ore (mean: 4800, SD: 1600 Bq m^−3^). The most extreme ^220^Rn value was in the Bolla shaft, which is a part of the old transport system leading into the other mines, whereas Bolla adit that lies above the shaft had relatively lower values of ^220^Rn and ^222^Rn since it is relatively short (50–60 m) and has openings in both ends leading to the draft pulling trough and dilution of airborne radionuclides. In mines running through redrock without ore, ^222^Rn levels were markedly higher but with large variation (mean: 2700, SD: 2300 Bq m^−3^) compared with in mines running through redrock with iron ore (mean: 900, SD: 300 Bq m^−3^). Levels were somewhat lower in FDC (Table 2) and the transition zone (means: 2000 (SD: 2400) and 2800 (SD: 2100) Bq m^−3^ for ^222^Rn and ^220^Rn). For all the ^220^Rn measurements, if the outlier (Bolla shaft) is disregarded, there is no correlation between these measurements and distance to the mine wall (*r* = −0.12, *p* > 06, Figure 5). There was, however, a near significant correlation between ^220^Rn measurements and bedrock ^232^Th levels (*r* = 0.40, *p* < 0.09, Figure 6), which was not affected by removal of the outlier in ^220^Rn. Levels of ^222^Rn were not correlated to the bedrock ^238^U measurements (*r* = −0.31, *p* > 0.2). All in all, the cold season dose rates calculated for inhalation during occupancy are high because of the ^222^Rn contribution in the Søve niobium mine, whereas in Mining Hill, it is the very high levels of ^220^Rn that are the main contributors (Tables 2).

**Table 2:** ^222^Rn and ^220^Rn activity concentrations in air measured with alpha track detectors in Fen legacy iron mines and in Søve Niobium mine with inhalation dose rate contributions (cDR) and combined cold season dose rate (ccDR) with gamma radiation. Abbreviations for bedrock: Fe-Dolomite carbonatite: FDC, Karup: K, redrock: R, redrock with iron: RI, transition zone: T, School mine: SM and søvite: S. Meters below surface given for Karup shaft localities. Control in School office in area covered by Holocene clay deposits (HCD).

Nr	Mine	Type	Zone	^222^Rn (Bq m^−3^)	^220^Rn (Bq m^−3^)	^222^Rn cDR (μSv h^−1^)	^220^Rn cDR (μSv h^−1^)	ccDR (μSv h^−1^)
1	SM	adit	FDC	1500	2400	5.0	3.8	10
2	SM	adit	T	960	3100	3.1	4.9	11
3	Lichtloch	shaft	T	1200	2500	3.7	4.1	10
4	Lichtloch	adit	T	<690	6600	2.2	11	15
5	Lichtloch	adit	R	1600	4700	5.2	7.5	18
6	Lichtloch	adit	RI	800	4100	2.6	6.6	18
7	Bolla	shaft	RI	880	45 000	2.9	72	85
8	AnneCat	adit^a^	T	1600	740	5.3	1.2	9.1
9	AnneCat	adit	T	560	2600	1.8	4.1	9.1
10	Western	Adit	T	6900	1100	22	1.8	28
11	K-40 m	shaft	RI	1300	1600	4.2	2.6	15
12	K-26 m	shaft	RI	1200	8100	4.0	13	26
13	K-17 m	shaft	RI	<600	2300	1.9	3.8	14
14	K-6 m	shaft	RI	<580	2900	1.9	4.6	11
16	Main adit	adit^b^	R	1700	5600	5.5	9.0	23
17	Main adit	adit	R	840	6900	2.7	11	23
18	Hov	adit	R	6600	3100	22	5.0	34
19	Bolla	adit	R	2500	3500	8.2	5.6	24
20	Søve	adit	S	5500	2200	18	3.5	22
15	Control	Office	HCD	<540	1900	1.7	3.0	4.8
16	Control	Office	HCD	<11	4700	0.1	7.5	7.6

a At base of shaft.

b Inclined adit.

Localities had different mine draft, ranging from hardly notable (estimated to be <0.2 m s^−1^ from the movement of plastic strings), to weak (estimated 0.2–0.3 m s^−1^) at most localities, but intermediate (estimated 0.5–1 m s^−1^) in adits of Søve (niobium mine), Bolla and main adit and shaft, to strong (estimated around 1.5–2 m s^−1^) in two Lichloch and one of the main adit localities. Deposition-based detectors were deployed at localities with intermediate or strong draft. The colder outside temperatures during the November deployment may have involved stronger chimney ventilation than during the deployment in December, when outside temperatures were higher.

The deposition-based TnP alpha track detector activity concentrations ranged from 88 to 760 Bq m^−3^ (median: 320, mean: 380, SD: 290) when not controlled for any mine draft airflow bias. After our correction of the bias, which probably reduced uncertainty, TnP ranged from 11 to 98 Bq m^−3^ (median: 42, mean: 49, SD: 37), still high levels. By comparison, when a correction for the wind bias of mine draft was done by correcting with the assumed airflow velocity divided by 0.02 m s^−1^ (lab conditions), corrected TnP values were reduced by another 67% compared with the correction of airflow bias done in this paper. In other words, the correction used here involves three times higher values than the most stringent way to correct for airflow because of mine draft (results not presented as this is only a factor difference).

Among the three assessed mines, the highest TnP level was found in the main adit (Table 3). In the main adit, the TnP dose rate contribution, when not controlled for airflow, contributes between half to two-thirds of the combined cold season dose rate. Consequently, when controlling for airflow bias on deposition, the TnP dose rate contributions are around one quarter of the combined cold season dose rate (Table 4). Compared with the equilibrium factor of *F* = 0.04 given by others for mines^(25)^, the inclined main adit (nr 16) and Hov adit (nr 18) have the most similar *F*-values when using TnP values controlled for airflow bias (Table 3). This suggests a significant bias from the airflow of mine draft at these localities, which was ~4-fold compared with our other localities with deposition detector deployment. By comparison, the main adit (nr 17), Bolla adit (nr 19) and the niobium mine (nr 20) have similar *F*-values as in the literature when their TnP values are not controlled for airflow bias. Also, the corresponding cold season dose rate contributions calculated from ^220^Rn levels in the inclined main adit (nr 16) and adit (nr 18) are similar to the corrected TnP cold season dose rate contributions (bold in Table 4). By comparison, the cold season dose rate contributions calculated from ^220^Rn levels in the main adit (nr 17), Bolla adit (nr 19) and in the niobium mine (nr 20) are similar to the non-corrected TnP cold season dose rate contributions (Table 4), suggesting there may not be much bias from mine draft at these mine localities. In total, between mines, among cold season dose rate contributions, gamma radiation contributes a fraction of 0.02–0.6, radon contributes a fraction of 0.33–0.95 and TnP a fraction of 0.1–0.25.

**Table 3:** Airborne TnP uncorrected with bias (BiasEECTn) and corrected for mine draft airflow bias (CorrEECTn) measured with deposition detectors at some Fen complex mine localities, and compared with measured ^220^Rn, the equilibrium factor, F, with the ones most similar to another mine study in bold

Nr	Mine	Type	Bedrock	BiasEECTn (Bq m^−3^)	CorrEECTn (Bq m^−3^)	Biased *F*	Corr *F*
16	Main adit	adit^a^	R w/I	760	98	0.134	**0.017**
17	Main adit	adit	R w/I	320	42	**0.047**	0.006
18	Hov	adit	R w/I	580	76	0.188	**0.024**
19	Bolla	adit	R, I, FDC	88	11	**0.025**	0.003
20	Søve	adit	F mix	150	19	**0.067**	0.009

a Inclined adit.

**Table 4:** For some Fen complex mine localities; uncorrected potentially air-flow-biased cold season dose rate contribution of TnP (BiascDR (μSv h^−1^)), the corresponding cold season dose contribution of TnP corrected for airflow of mine draft (CorrcDR (μSv h^−1^)) and combining also with gamma and radon dose rate, the combined cold season dose rate of these mine localities at minimum (Min ccDR) and maximum (Max ccDR), depending on whether airflow correction has been performed. Most probable values in bold.

Nr	Mine	Type	Bedrock	BiascDR (μSv h^−1^)	CorrcDR (μSv h^−1^)	Max ccDR (μSv h^−1^)	Min ccDR (μSv h^−1^)
16	Main adit	adit^a^	R w/I	30	**3.9**	44	**17**
17	Main adit	adit	R w/I	**13**	1.7	**25**	14
18	Hov	adit	R w/I	23	**3.0**	52	**32**
19	Bolla	adit	R, I, FDC	**3.5**	0.46	**22**	19
20	Søve	adit	F mix	**5.8**	0.76	**24**	19

a Inclined adit

## Discussion

In this study, gamma radiation measurements were made perpendicularly to the roof at some distance. These results are therefore not entirely representative in relation to the gamma dose rates received by humans during occupancy in the mine. The dose rate from gamma radiation measurements should therefore be verified with a gamma dosemeter suitable for gamma from all directions. Moreover, even though the assumed secular equilibrium may hold for rock not exposed to weathering^(33)^, deviations may occur where there is leaching of uranium and radium isotopes with weathering through fissures^(34, 35)^. Since gamma measurements target progeny, any large deviations from secular equilibrium would involve even higher levels of progenitor radionuclides than measured. The large difference in gamma from the Mining Hill localities within redrock (with or without iron ore) compared with the transition zone and the FDC zone is clearly because of the amount of redrock and its level of ^232^Th. The lower value of the Søve niobium mine is because of the Fenite mix bedrock measured here. The niobium mine elsewhere runs through søvite. Søvite is usually not so enriched in ^232^Th but rather ^238^U, which emits much less gamma but explains the high levels of ^222^Rn in Søve.

The levels of ^222^Rn and ^220^Rn within the assessed mines during the cold season with rising mine draft are relatively high and are clearly relevant regarding dose rate contribution (Table 2). The levels seem to be related to the type of bedrock each mine runs through and the bedrock level of primordial radionuclide. Clearly, levels of ^220^Rn are higher in mines with redrock and iron ore, lower when redrock has less or no ore, and even less in the transition and FDC zones. This is supported by the near significant correlation between ^220^Rn and bedrock levels of ^232^Th. The ventilation pattern is probably also important and may in places with significant draft explain why these correlations are not stronger for ^220^Rn. However, with a mine draft of 0.1 m s^−1^ and no exhalation, a parcel of air would in five half-lives only move 27 meters, meaning that any high ^220^Rn levels must originate from nearby bedrock with high levels of progenitor radionuclides. By comparison, ^222^Rn has a much longer half-life (3.8 d), which enables transport for a much longer time and can explain the lacking correlations of ^222^Rn with bedrock progenitor levels. For example, air will in 24 h be moved 8640 meters by a mine draft of 0.1 m s^−1^, which involves little decay for ^222^Rn. In the niobium mine, there are large bedrock heterogeneities in radionuclide distribution, as this mine runs through various bedrock like FDC and søvite. In addition, søvite has a heterogenous distribution of ^238^U that may be especially enriched in patches of pyrochlore^(21)^. These heterogeneities are not apparent in the single gamma spectrometric measurement from the niobium mine, but with mine draft, which was observed as intermediate, it may explain the high ^222^Rn level measured. In the Fen legacy iron mines, mine draft must also be significant in places, as both ^220^Rn and ^222^Rn have been recorded at high levels in air ventilating from the mines in Mining Hill with important local outdoor impact^(1, 28, 36)^. The observed differences in the levels of these gases between iron legacy mine localities thus probably reflects the differences in both bedrock radionuclide levels and mine draft. However, the levels of these gasses at the different localities and the whole pattern may be different with the opposite direction of mine draft in summer, which is characterised by a sinking draft.

By comparison, another legacy metalliferous (wolframite) mine had much lower levels of both airborne radionuclides, with ^222^Rn ranging from 120 to 150 Bq m^−3^ and ^220^Rn ranging from 270 to 960 Bq m^−3^, and bedrock levels of primordial radionuclides of 140 Bq kg^−1^ of ^226^Ra and 120 Bq kg^−1^ of ^232^Th^(25)^. That study involved dose rates of 0.3, 1.2 and 0.5 μSv h^−1^ from RnP, TnP and gamma, respectively. As mentioned previously, there was significant mine draft, but the study did not assess bias on the measured TnP levels, which ranged from 8 to 10 Bq m^−3^^(25)^, even though uncertainties in these measurements were in the order of 10 Bq kg^−1^ and a bias cannot be excluded. The equilibrium factor F in the Kleinschmidt *et al*.’s^(25)^ study is similar to the *F*-values of three localities of the study at hand with TnP levels not corrected for wind bias (one main adit location, Bolla and Søve mines) but similar to the *F*-values of two localities with TnP values corrected for wind bias (inclined main adit and Hov adit). The difference corresponds well with the approximate 4-fold difference in felt mine draft between these mine localities (uncorrected versus corrected). This suggests a bias from airflow, at least at higher airflow velocities, as suggested by previous studies^(28)^. The characterisation of the natural ventilation of the Mining Hill mines strongly suggests a mainly seasonal chimney ventilation^(1)^, which gives the seasonal mine draft directions but no prediction of the magnitude of draft. Such natural ventilation can be large^(37–41)^. There is therefore much potential for different residence times for ^222^Rn, ^220^Rn and potentially different disequilibrium factor values.

Regardless the size of the potential bias on deposition-based TnP detectors, in the Mining Hill mines there is significant gamma radiation, which dose rate contribution must be considered for any occupancy. Notice, however, that because of the lower levels of ^232^Th, gamma radiation dose rate was lower in the FDC zone and especially much lower in the søvite zone. For Mining Hill localities and especially the niobium Søve mine, the cold season dose rate contribution of ^222^Rn is significant and must also be considered. Notice that we have applied the UNSCEAR DCFs, whereas ICRP DCFs^(42)^ for the ^222^Rn dose rate contribution would near double in significance and the ^220^Rn dose rate contribution would triple. From the most probable equilibrium factors calculated, either for potentially biased or for corrected TnP values (Tables 3 and 4), we assume a combined cold season dose rate of ~17–24 μSv h^−1^. The most probable TnP cold season dose rate contribution being from a tenth to a fourth of the combined cold season dose rate would with ICRP DCFs change to constituting from a third to over half of the combined cold season dose rate. Since new mining activities for REE and ^232^Th are being planned for the Fen complex, exposure pathways for both the cold and warm season dose rate contributions are needed to consider occupancy. Exposure pathways and dose rate contributions should therefore also be assessed during the warm season and during the transient seasons, which could have diurnal changes in draft direction.

Unfortunately, measurements of TnP were not done in the School adit. We must stress that only this one of our localities was situated truly within the FDC bedrock unit, and because it was situated in a dead-end adit, which is anomalously dry and not ventilated, it is not comparable to the other localities neither in bedrock nor ventilation. Hence, other localities within the FDC unit must be investigated to validate this result. Given the levels of ^222^Rn and ^220^Rn at the FDC location, the cold season dose rate contributions of these gasses are probably less significant than within the redrock zone. However, it was noted within the FDC zone adit a much higher level of fine airborne dust, and since the DCF’s of inhalation of the ^238^U and ^232^Th associated with such dust is several orders of magnitude larger than for progeny of ^222^Rn and ^220^Rn, the real combined cold season (and possibly warm season) dose rates in mine within FDC may be larger and should be assessed in the future.
